# A prognostic hypoxia gene signature with low heterogeneity within the dominant tumour lesion in prostate cancer patients

**DOI:** 10.1038/s41416-022-01782-x

**Published:** 2022-03-24

**Authors:** Unn Beate Salberg, Vilde Eide Skingen, Christina Sæten Fjeldbo, Tord Hompland, Harald Bull Ragnum, Ljiljana Vlatkovic, Knut Håkon Hole, Therese Seierstad, Heidi Lyng

**Affiliations:** 1 Department of Radiation Biology, Institute for Cancer Research, Oslo University Hospital, Radiumhospitalet, Oslo Norway; 2 Institute of Clinical Medicine, University of Oslo, Oslo, Norway; 3 Department of Physics, University of Oslo, Oslo, Norway; 4 Department of Haematology and Oncology, Telemark Hospital Trust, Skien, Norway; 5 Department of Pathology, Oslo University Hospital, Oslo, Norway; 6 Division of Radiology and Nuclear Medicine, Oslo University Hospital, Oslo, Norway

**Keywords:** Prostate cancer, Tumour biomarkers, Prognostic markers, Tumour heterogeneity, Cancer microenvironment

## Abstract

**Background:**

Gene signatures measured in a biopsy have been proposed as hypoxia biomarkers in prostate cancer. We assessed a previously developed signature, and aimed to determine its relationship to hypoxia and its heterogeneity within the dominant (index) lesion of prostate cancer.

**Methods:**

The 32-gene signature was assessed from gene expression data of 141 biopsies from the index lesion of 94 patients treated with prostatectomy. A gene score calculated from the expression levels was applied in the analyses. Hypoxic fraction from pimonidazole immunostained whole-mount and biopsy sections was used as reference standard for hypoxia.

**Results:**

The gene score was correlated with pimonidazole-defined hypoxic fraction in whole-mount sections, and the two parameters showed almost equal association with clinical markers of tumour aggressiveness. Based on the gene score, incorrect classification according to hypoxic fraction in whole-mount sections was seen in one third of the patients. The incorrect classifications were apparently not due to intra-tumour heterogeneity, since the score had low heterogeneity compared to pimonidazole-defined hypoxic fraction in biopsies. The score showed prognostic significance in uni-and multivariate analysis in independent cohorts.

**Conclusions:**

Our signature from the index lesion reflects tumour hypoxia and predicts prognosis in prostate cancer, independent of intra-tumour heterogeneity in pimonidazole-defined hypoxia.

## Background

Improved biomarkers to select patients with aggressive prostate cancer are required, since current clinical markers are not sufficient to identify those in need of intensified therapy [1]. Tumour hypoxia is associated with aggressive disease and increased risk of biochemical recurrence after radical prostatectomy or radiotherapy [2–5]. Hypoxia is therefore a potential biomarker and target for therapeutic intervention. Gene expression signatures measured in tumour biopsies have emerged as exciting hypoxia biomarkers [6] and promising candidates have been developed for prostate cancer [7, 8]. The signatures measure the transcriptional state of cells based on a set of hypoxia-related genes determined for each cancer type. In addition to the absence or presence of hypoxia, this information can indicate resistance mechanisms that are at play in the tumour. Such signatures may therefore help decision about molecular targeting drugs to be used in an adjuvant setting, making them attractive as biomarkers [9, 10]. However, the signatures originate from a small tissue sample and may fail to reflect the most aggressive feature of the disease due to intra-tumour heterogeneity [11]. This heterogeneity is a major concern for the implementation of biopsy-based biomarkers in the clinic [12].

The vast majority of prostate cancer patients present with several tumour lesions within the prostate gland [13]. Considerable differences in gene expression and genetic aberrations exist both within and between lesions that may impact molecular classification of individual patients [14–22]. Introduction of magnetic resonance imaging (MRI)-directed biopsy sampling of suspected prostate cancer based on the recent prostate imaging reporting and data system (PIRADS), will most likely improve patient classification [23, 24]. By this advancement, biopsies can with high accuracy be collected from the assumed index lesion, defined as the dominant intra-prostatic lesion according to clinical markers like tumour stage and size. However, the index lesion is often large compared to biopsy size and may show heterogeneity in prognostic features like hypoxia. In accordance with this, large oxygen gradients within prostate tumours have been demonstrated by immunostaining of histological sections, using the hypoxia marker pimonidazole [5, 25]. To understand the potential of gene signatures as hypoxia biomarkers, their heterogeneity within the index lesion and how this heterogeneity affects their ability to predict disease aggressiveness have to be clarified.

We have developed a 32-gene signature with potential as hypoxia biomarker in prostate cancer, as described in Ragnum et al. [7]. The Ragnum-signature was constructed by combined analysis of gene expression profiles and pimonidazole-staining in biopsies from the index lesion of 39 patients. The signature was shown to correlate with clinical markers of tumour aggressiveness in two independent prostate cancer cohorts. In the present work, we utilised whole-mount sections and multiple biopsies from an extended prostatectomy cohort of 114 patients. We aimed to determine the ability of the Ragnum-signature to reflect hypoxia in the index lesion and correctly classify tumours as more or less hypoxic. Biopsy location within the index lesion was verified from the prostatectomy specimens. Moreover, hypoxic fraction of the entire lesion, as defined by pimonidazole, was used as a reference standard. This parameter represents the tissue fraction with an oxygen level below about 10 mmHg (1.3% O_2_) [26]. We further used paired biopsies from the index lesion to assess the intra-tumour heterogeneity of the signature, which we compared with the pimonidazole-defined heterogeneity and with the classification output. A digital histopathology platform was developed to quantify hypoxic fraction from pimonidazole-stained sections. Such platform is a resource that has been lacking in previous work on prostate cancer [5, 7, 27], and facilitated reproducible comparison of hypoxic fraction across samples and with the gene signature data.

## Patients and methods

### Patient cohort

The study included 114 prostate cancer patients referred to robot-assisted laparoscopic radical prostatectomy (RALP) at Oslo University Hospital between October 2011 and May 2016. All patients were enrolled in the FuncProst study (NCT01464216). Except for three low-risk patients, all had intermediate- or high-risk disease according to the D’Amico risk classification [28] (Table 1).

**Table 1:** Prostate cancer cohort.

Clinical markers	Patients (*n* = 114)	Patients with two biopsies (*n* = 52)
Age (years)	65 (45–76)	66 (45–76)
PSA (μg/l)	8.8 (2–145)	10.5 (2.2–145)
D’Amico risk classification		
Low	3 (3)	0 (0)
Intermediate	54 (48)	22 (43)
High	56 (49)	29 (57)
Lymph node status		
Positive	11 (10)	9 (18)
Negative	100 (90)	41 (82)
Pathological tumour stage		
2	36 (32)	9 (18)
3	76 (67)	41 (80)
4	1 (1)	1 (2)
ISUP grade group		
1	9 (8)	1 (2)
2	47 (42)	14 (27)
3	27 (24)	18 (35)
4	17 (15)	10 (19)
5	13 (11)	8 (17)

Patients with two biopsies were a subgroup of the cohort with 114 patients. Age and PSA are described as median value (range). Remaining data are described as number of patients (%).

Most patients (*n* = 102; Supplementary Fig. S1) received the hypoxia marker pimonidazole hydrochloride (Hypoxyprobe Inc., Burlington, MA, USA) intravenously or orally at a dose of 500 mg per m^2^ 13–25 h prior to surgery, as previously described [5]. A three-armed robotic da Vinci surgical system (Intuitive Surgical, Sunnyvale, CA, USA) was used to perform RALP [29]. Guided by palpation, preoperative biopsy results and multiparametric MRI, the prostate was cut into two halves where the index lesion was assumed to be located [7]. Two 6 mm punch biopsies were taken from the anticipated tumour site, snap-frozen in liquid nitrogen and stored at −80 °C. The prostate specimen was fixed in 10% buffered formalin for minimum 48 h. Grossing was performed according to standard protocols, where total prostate with seminal vesicles was embedded [30]. The apex and base of the prostate were cut as sagittal sections, whereas the remaining body was cut into 3–4 mm transverse slices and prepared as whole-mount sections. Haematoxylin and eosin (HE)-stained whole-mount sections of 5 µm thickness were used for histopathological staging and grading, applying the 7th edition of TNM classification for staging [31] and the grading system suggested by the International Society of Urological Pathology (ISUP) in 2014 [32].

Tumour lesions and location of each biopsy were identified by examination of macro images and MR images, guided by HE-stained whole-mount sections and anatomical landmarks like seminal vesicles, ejaculatory ducts, relative distance from the prostatic capsule and midline (Supplementary Fig. S2). For patients with multiple lesions, the index lesion was defined based on, in descending order, pathologic tumour (T) stage, Gleason score and tumour size [33]. Only biopsies from the index lesion with at least 75% malignant glands and less than 50% stroma in HE-stained sections were used, reducing confounding effects caused by possible differences in cellular composition across samples. Totally 142 biopsies from 95 patients, including 83 patients who received pimonidazole, fulfilled these criteria (Supplementary Fig. S1). These biopsies were used to assess hypoxia by the pimonidazole-defined (*n* = 129) and gene-defined (*n* = 141) assays.

### Pimonidazole-defined hypoxia assay

Immunohistochemistry was performed to visualise hypoxia in histological sections by using monoclonal mouse antibody for pimonidazole (1:100, whole-mount sections; 1:50, biopsy sections; Hypoxyprobe Inc), as previously described [5, 7]. Out of 102 patients who received pimonidazole, whole-mount sections from 100 patients and totally 129 biopsy sections from 83 patients were available for immunohistochemistry (Supplementary Fig. S1). The biopsy sections were from 46 biopsy pairs from the same lesion and 37 single biopsies. Antigen retrieval was performed using PT-Link (Dako, Glostrup, Denmark) and EnVision TM target retrieval solution (Dako). The immunohistochemistry protocol was optimised for the formalin fixated whole-mount sections and the fresh frozen biopsy sections separately, to achieve satisfactory dynamics and stability in the staining intensities. A high pH was applied for the whole-mount sections, while sections from fresh frozen biopsies were boiled in citrate buffer. Haematoxylin was used as counterstain.

A digital histopathology platform was developed in Matlab to assess quantitative measures of hypoxic fraction based on pimonidazole-staining (Supplementary Methods S1). Route of pimonidazole administration or time from administration to prostate dissection had no influence on staining intensity or pattern [5]. All sections were therefore analysed together. The whole-mount and biopsy sections were imaged by a NanoZoomer 2.0-HT and NanoZoomer-XR slide scanner (Hamamatsu, Hamamatsu City, Japan), respectively. In images of the whole-mount sections, the index lesion was outlined by comparing the pimonidazole-stained section with the corresponding HE-stained section, and hypoxic fraction of the entire lesion (HF_W-m_) was calculated. For biopsies, hypoxic fraction (HF_Biopsy_) was calculated based on the entire section. Hypoxia scores ranging from 1–5, as determined by two experienced uro-pathologists for all 100 whole-mount sections and 38 biopsy sections, were available from previous work [5, 7]. These pathologist’s scores were used to define a staining intensity threshold for segmentation of hypoxic regions and to evaluate the performance of the digital quantification (Supplementary Methods S1). Cohen’s analysis of the hypoxia scores in biopsies showed a good inter-observer agreement for the two pathologists, with a kappa-coefficient (κ) of 0.80 [7]. HF_W-m_ and HF_Biopsy_ were used as a reference standard for hypoxia in analyses against gene data.

### Gene-defined hypoxia assay

Gene expression profiling was carried out for 141 biopsies from 94 patients (47 biopsy pairs and 47 single biopsies; Supplementary Fig. S1), using Illumina bead arrays HT-12 v4 (Illumina Inc., San Diego, CA, USA) with about 47 300 transcripts. Total RNA was isolated from a piece of each biopsy (10 slices × 20 μm), using RNeasy MiniKit (*n* = 45) or miRNeasy MiniKit (*n* = 96) (Qiagen, Hilden, Germany). All samples had an RNA integrity number (RIN) of 6 or above, as assessed by the Agilent 2100 Bioanalyzer (Agilent Technology, Santa Clara, CA, USA). Complementary RNA (cRNA) was synthesised, labelled and hybridised to the arrays. Signal extraction and quantile normalisation were performed using the software provided by the manufacturer (Illumina Inc.). Gene expression profiles of the RNeasy-derived samples were transformed by centring the median expression value of every probe to the median value of the miRNeasy samples, as in previous work [34]. Probe re-annotation was performed using the R package org.HS.eg.db (v3.7.0) [35]. The gene expression data have been deposited to the Gene Expression Omnibus (GEO) repository (GSE178631).

The Ragnum-signature developed in previous work on a subgroup of 39 patients in this cohort [7] was used. The 32 signature genes were identified in the original study because they showed a significant correlation between their expression level and the pathologist’s hypoxia score assessed in pimonidazole-stained biopsies [7]. One probe per gene was selected based on the highest interquartile range. A gene score was calculated for each patient by averaging the median-centred, log_2_-transformed expression level of the 32 genes [7]. This score was used as a measure of the gene signature. For analyses based on individual genes, log_2_-transformed expression levels were used. Time from complete dissection of the prostate during surgery to snap-freezing of biopsies (ischemia time) had no influence on the gene score [7].

### Intra-tumour heterogeneity

Intra-tumour heterogeneity in HF_Biopsy_, gene score and the expression level of individual signature genes was compared for patients where two biopsies from the index lesion were available (Supplementary Fig. S1). All pathological tumour stages and ISUP grade groups were represented in this sub-cohort (Table 1). Two parameters describing the heterogeneity were calculated for 41 biopsy pairs with both HF_Biopsy_ and gene score data; the relative difference and the correlation between two biopsies from the same lesion. The relative difference was derived as the difference in HF_Biopsy_, gene score or gene expression level between the two biopsies divided by the overall range (the maximum value minus the minimum value of the 82 biopsies). The correlation was assessed by Pearson correlation analysis of scatter plots where the highest biopsy value in a pair was plotted against the lowest value.

### External gene expression datasets

Gene expression data from the primary prostate tumour of three independent prostatectomy cohorts were used to investigate the relationship between the Ragnum-signature and clinical outcome. The cancer genome atlas (TCGA) dataset [36] included RNA sequencing data from Illumina HiSeq RNAseq v2 and disease-free survival status of 491 patients (TCGA-PRAD). In addition, pathological T-stage and ISUP grade group were extracted for multivariate analysis. The Cambridge (GSE70768) and Stockholm (GSE70769) datasets had gene expression data from Illumina bead arrays HT-12 v4 and biochemical recurrence status of 111 and 92 patients, respectively [37]. Biochemical recurrence was defined according to the European Guidelines as a persistent rise in prostate specific antigen (PSA) above 0.2 ng/ml or triggered by salvage radiotherapy. All data were obtained in a single fresh frozen biopsy from the prostatectomy specimens [36, 37]. Pathology examination showed that tumour cell fraction was above 20% in most cases. Expression data of all 32 signature genes were available and used for calculation of the gene score for each patient.

### Statistics

One biopsy per patient was used to assess associations between HF_W-m_, HF_Biopsy_ and gene score and to compare these parameters between groups of patients with different clinical markers. For patients with two biopsies, the biopsy collected first was selected as the representative one (*T1*, Supplementary Fig. S2B, C). Mann-Whitney U-test was applied on data comparing two groups. When comparing two groups of patients for each of three clinical markers (lymph node status, ISUP grade, pathological T-stage), *P*-values adjusted for multiple testing by Benjamini-Hochberg correction [38] were reported (adj *P*), in addition to nominal *P*-values. Correlation between data was assessed by Pearson’s correlation. Cox uni- and multivariate proportional-hazards (PH) analysis was performed to evaluate prognostic significance of the gene score. Assumptions of PHs were tested graphically using log-minus-log plots (not shown). Kaplan–Meier curves were compared using log-rank test. All analyses were performed in R v4.0.2. Probability values of *P* < 0.05 were considered statistically significant.

## Results

### Relationship between tumour aggressiveness and hypoxic fraction of the index lesion

**Fig. 1:**
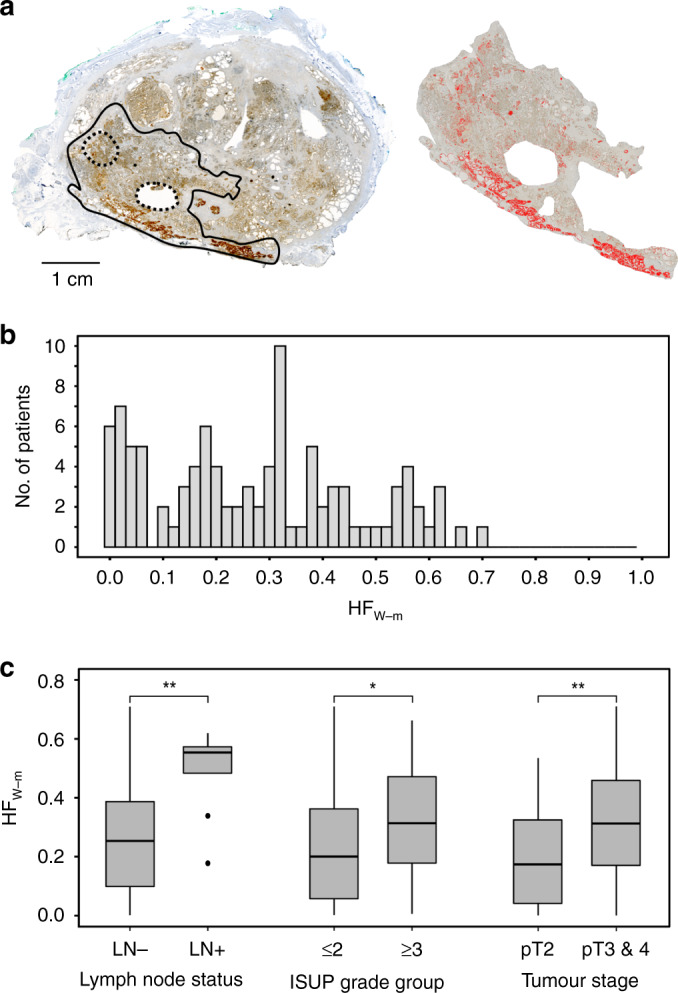
Pimonidazole-defined hypoxia in the index lesion in relation to tumour aggressiveness. **a** Pimonidazole immunostained whole-mount section (left) and the part of the section showing index lesion (right). Solid and dotted lines in the whole-mount section indicate the index lesion and location of punch biopsies, respectively. The hypoxic, pimonidazole positive regions segmented by digital histopathology and used to calculate HF_W-m_ are indicated in red (right). **b** Frequency distribution of HF_W-m_ for 100 patients. **c** Boxplots of HF_W-m_ for patients with positive and negative lymph node (LN) status (*n* = 99), ISUP grade group of ≤2 and ≥3 (Gleason grade ≤3 + 4 and ≥4 + 3) (*n* = 100) and pathological tumour stage T2 and T3 or T4 (*n* = 100). The boxes extend from the first to the third quartile with the median value indicated. Significant difference between groups by Mann-Whitney U-test is indicated, **(*P* < 0.01) and *(*P* < 0.05).

A digital procedure to quantify hypoxic fraction defined from pimonidazole-staining of whole-mount sections was established (Fig. 1a). By this procedure, a highly significant correlation between HF_W-m_ and the pathologist’s hypoxia score in 100 patients was obtained (r = 0.78; *P* < 0.001, Supplementary Methods S1). HF_W-m_ varied considerable across patients, ranging from 0 to 0.71 with a median of 0.27 (Fig. 1b). Moreover, a high HF_W-m_ was associated with clinical markers of aggressive disease (Fig. 1c), including positive lymph node status (*P* = 0.002; adj *P* = 0.006), ISUP grade group of 3 or above (*P* = 0.042; adj *P* = 0.042) and advanced pathological tumour stage of T3 or T4 (*P* = 0.004; adj *P* = 0.006), in accordance with our previous work based on the pathological hypoxia score [5]. These data indicated how much hypoxic fraction of the index lesion differed between clinically relevant patient groups, which was useful for evaluation of the intra-tumour heterogeneity data. The difference in median HF_W-m_ was 0.30 for patients with positive and negative lymph node status and 0.11 for patients with high and low ISUP grade (Fig. 1c). For patients with more advanced and less advanced tumour stage, this difference was 0.14.

### Assessment of tumour aggressiveness and hypoxia from a single biopsy

**Fig. 2:**
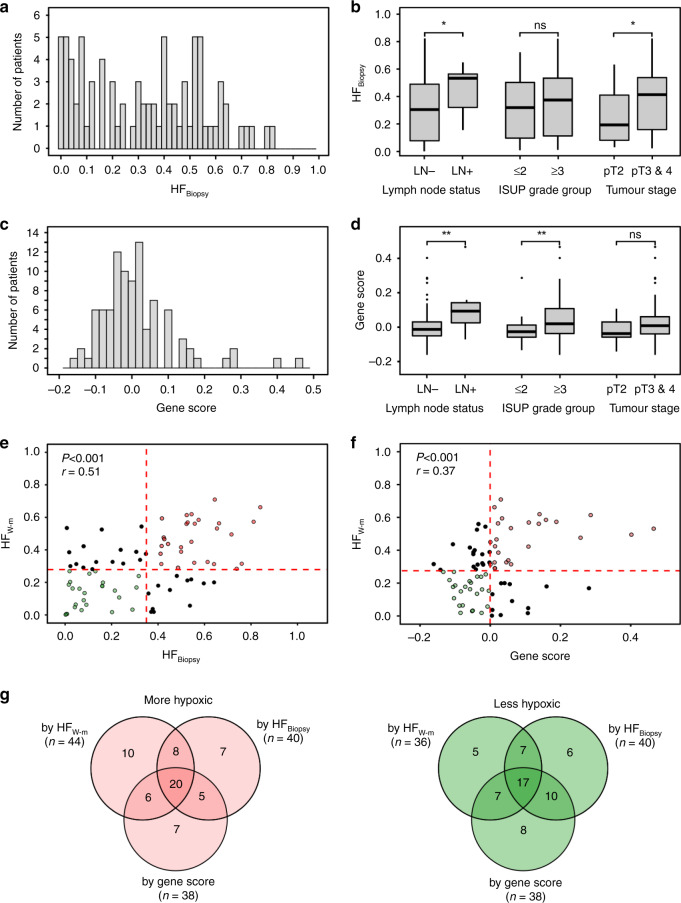
Pimonidazole- and gene-defined hypoxia in biopsies in relation to tumour aggressiveness and hypoxia. **a** Frequency distribution of HF_Biopsy_ for 83 patients. **b** Boxplots of HF_Biopsy_ for 83 patients with positive and negative lymph node (LN) status, ISUP grade group of ≤2 and ≥3 (Gleason grade ≤3 + 4 and ≥4 + 3) and pathological tumour stage T2 and T3 or T4. **c** Frequency distribution of gene score in a single biopsy from 94 patients. **d** Boxplots of gene score for patients with positive and negative lymph node (LN) status (*n* = 92), ISUP grade group of ≤2 and ≥3 (Gleason grade ≤3 + 4 and ≥4 + 3) (*n* = 93) and pathological tumour stage T2 and T3 or T4 (*n* = 93). **e** Association between HF_Biopsy_ and HF_W-m_ for 81 patients with both datasets available. **f** Association between gene score and HF_W-m_ for 80 patients with both datasets available. **g** Venn-diagrams showing the overlap in classification of 80 patients with HF_W-m_, HF_Biopsy_ and gene score available. The median values indicated in **e**, **f** were used as classification cutoffs. Results are shown for tumours classified as more hypoxic (*n* = 44, left) and less hypoxic (*n* = 36, right) by HF_W-m_, separately. **b**, **d** The boxes extend from the first to the third quartile with the median value indicated. Significant difference between groups by Mann-Whitney U-test is indicated, **(*P* < 0.01) and *(*P* < 0.05). **e**, **f** The red dotted lines indicate separation between more and less hypoxic tumours based on the median value of the distribution in panel **b** and panels **a**, **c**. Pearson correlation coefficient (r) and *P*-value are indicated.

In analysis of a single biopsy from each tumour, we first determined how well HF_Biopsy_ and gene score reflected tumour aggressiveness. HF_Biopsy_ showed a similar distribution as HF_W-m_ and ranged from 0 to 0.82 with a median of 0.34 (Fig. 2a). A significant association with lymph node status (*P* = 0.023; adj *P* = 0.069) and pathological tumour stage (*P* = 0.03; adj *P* = 0.045), but not with ISUP grade group, was found (Fig. 2b). The gene score showed a normal like distribution (Fig. 2c). The score was higher in patients with positive lymph node status (*P* = 0.003; adj *P* = 0.0045) and ISUP grade group of 3 or above (*P* = 0.0016; adj *P* = 0.0048), while a borderline difference was found for pathological tumour stage (*P* = 0.058; adj *P* = 0.058) (Fig. 2d). The association between gene score and clinical markers as reported in our previous work [7], was therefore valid in the extended cohort of patients. Moreover, this association was retained in analysis based only on biopsies that were not included in our previous work (Supplementary Fig. S3A). Taken together, the gene score and HF_W-m_ seemed to show a similar, strong association to tumour aggressiveness, while the association was slightly weaker for HF_Biopsy_.

We further determined the ability of HF_Biopsy_ and gene score to reflect hypoxia in the index lesion. Both parameters were significantly correlated with HF_W-m_ in analysis of continuous data (r = 0.51; *P* < 0.001; r = 0.37, *P* = 0.001; Fig. 2e, f). The gene score also showed a strong correlation with HF_W-m_ compared to most of the individual signature genes (Supplementary Table S1) and compared to a previously published hypoxia gene signature (Supplementary Table S2), which was developed in prostate cancer by Yang et al. [8]. Notably, the correlation with HF_W-m_ for the Yang-signature was on the borderline of significance, whereas no correlation was found between the Yang- and the Ragnum-signature (Supplementary Table S2). A significant correlation was further found between our gene score and HF_Biopsy_ (r = 0.39; *P* < 0.001; Supplementary Fig. S4A), in accordance with previous work on a subgroup of the patients [7]. In line with the results presented in Supplementary Fig. S3A, this correlation was retained when based on biopsies that were not included in previous work (Supplementary Fig. S3B).

For dichotomous classification, the median value based on all patients (Figs. 1b, 2e, f) was applied as cut-off to define hypoxia status for each parameter. Out of 80 patients with HF_W-m_, HF_Biopsy_ and gene score available, 44 patients had more hypoxic tumour and 36 had less hypoxic tumour according to HF_W-m_. Totally 20 (57%) tumours were classified with more hypoxia and 17 (47%) tumours with less hypoxia by all three assays (Fig. 2g, h). Moreover, 52 (65%) tumours were correctly classified by HF_Biopsy_ and 50 (63%) by the gene score according to HF_W-m_ (Fig. 2g). Approximately the same overlap with HF_W-m_ was found for other cut-off values. In particular, by using a cut-off defined by the 67% percentile, which is about the expected recurrence rate for patients with intermediate or high-risk prostate cancer following prostatectomy [39, 40], the overlap was 69% (HF_Biopsy_) and 65% (gene score) (Supplementary Fig. S4B). Thus, although totally different assays, incorrect classifications were seen for about one third of the patients for both HF_Biopsy_ and gene score.

### Heterogeneity in hypoxic fraction and gene score within the index lesion

**Fig. 3:**
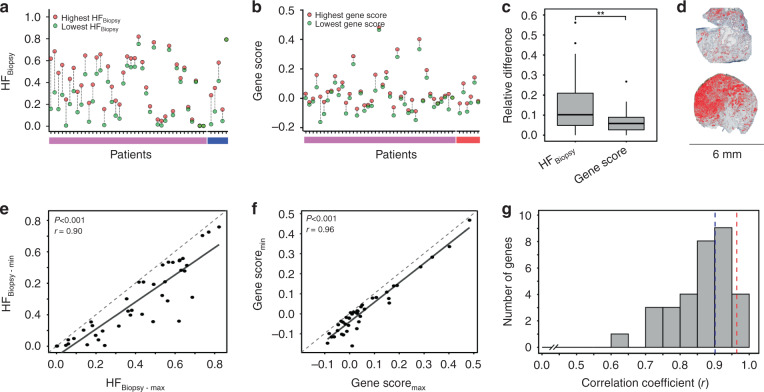
Heterogeneity in pimonidazole- and gene-defined hypoxia within the index lesion. **a** HF_Biopsy_ for biopsy pairs collected from 46 patients. **b** Gene score for biopsy pairs collected from 47 patients. **c** Boxplot of relative difference between two biopsies from the same index lesion for HF_Biopsy_ and gene score (*n* = 41 patients). The boxes extend from the first to the third quartile with the median value indicated. Significant difference between groups by Mann-Whitney U-test is indicated, **P = 0.002. **d** Pimonidazole-stained sections of a biopsy pair collected from the index lesion, one biopsy with low (left) and another with high (right) HF_Biopsy_. The hypoxic, pimonidazole positive regions segmented by digital histopathology and used to calculate HF_Biopsy_ are indicated in red. **e** Scatter plot of the highest HF_Biopsy_-value in a biopsy pair *versus* the lowest value. **f** Scatter plot of the highest gene score in a biopsy pair *versus* the lowest value. **g** Frequency distribution of Pearson correlation coefficient (r) for 32 signature genes, shown in intervals of 0.05. The dotted lines indicate the coefficient of the gene score (r = 0.96, red) and HF_Biopsy_ (r = 0.90, blue). **a**, **b** The patients are sorted based on decreasing difference in HF_Biopsy_. *X*-axes indicate patients included in both (**a**) and (**b**) (pink), patients with only HF_Biopsy_ (blue, **a**) and patients with only gene score (**b**, red). **e**, **f** Pearson correlation coefficient (r) and *P*-value are indicated. Stippled line—1:1 relationship, solid line—linear regression line.

To investigate whether incorrect classification by the pimonidazole- and gene-defined assays could be a consequence of intra-tumour heterogeneity, we analysed two biopsies from each lesion of 46 (HF_Biopsy_) and 47 (gene score) patients. There was a large difference in HF_Biopsy_ of up to 0.46 for some biopsy pairs and above 0.10 in more than half of the pairs (Fig. 3a). Differences were also seen in the gene score when comparing two biopsies from the same lesion (Fig. 3b), ranging from 0 to 0.17 with a median of 0.04. In many cases, the difference in HF_Biopsy_ or gene score within the index lesion was therefore larger than between groups of patients with different clinical markers of aggressive disease (Figs. 1d and 2b, d).

The intra-tumour heterogeneity in HF_Biopsy_ and gene score was compared based on the difference between the two biopsies in a pair, using 41 patients with both datasets available. The relative difference was significantly lower for the gene score than for HF_Biopsy_ (*P* = 0.002; Fig. 3c). The large difference in pimonidazole-defined hypoxia was clearly visible in histological sections of many biopsy pairs (Fig. 3d). In scatter plots, where the highest biopsy value in a pair was plotted against the lowest value, a stronger correlation was found for the gene score (r = 0.96) than for HF_Biopsy_ (r = 0.90). Moreover, the scatter seemed to be approximately constant over the range of measured values without any higher heterogeneity at the highest values. The gene score further showed a lower relative difference and a stronger correlation than most signature genes (Fig. 3g; Supplementary Table S1). These results indicated a high reproducibility of the gene score within the index lesion. Moreover, incorrect classification of some tumours according to HF_W-m_ appeared to be caused by intra-tumour heterogeneity for HF_Biopsy_ but not for the gene score.

### Gene score in relation to clinical outcome in external datasets

**Fig. 4:**
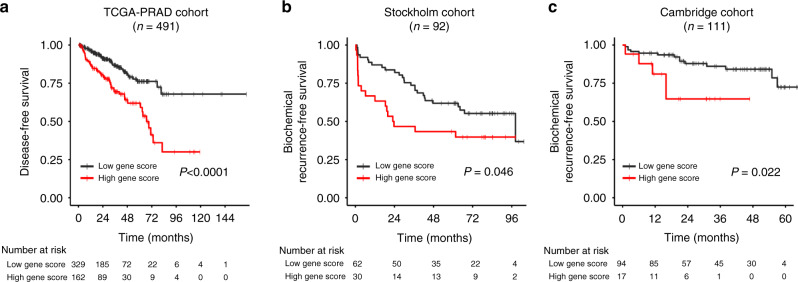
Prognostic impact of gene-defined hypoxia in external prostatectomy cohorts. Kaplan–Meier curves for disease-free survival of 491 patients in the TCGA-PRAD cohort (**a**), and biochemical recurrence-free survival of 92 patients in the Stockholm cohort (**b**) and 111 patients in the Cambridge cohort (**c**). The patients were classified with a high (red curve) or a low (black curve) gene score. **a**, **b** 33% of the patients were classified to the group with a high gene score, **c** 15% of the patients were classified to this group. *P*-values in log-rank test and number of patients at risk are indicated.

The potential of the gene score as biomarker when based on only one biopsy per patient was further evaluated by investigating its prognostic impact in three publicly available datasets; the TCGA-PRAD, Stockholm and Cambridge prostatectomy cohorts. Cox univariate analysis showed a significant correlation between the continuous gene score and treatment outcome for all cohorts (*P* < 0.00001, TCGA-PRAD; *P* < 0.001, Stockholm; *P* = 0.016, Cambridge). The cut-off for classification of patients into groups with a high or a low gene score was determined by considering all possible gene score values. A significant difference in outcome between the patient groups was found for all cohorts (Supplementary Fig. S4). For the TCGA-PRAD and Stockholm cohorts, significant results were achieved for a range of percentiles, including the 67% percentile where about one third of the patients were classified with a high gene score (*P* < 0.0001, TCGA-PRAD; *P* = 0.046, Stockholm; Fig. 4a, b). By using a higher cut-off that classified 15% of the patients in the high score group, a significant association with biochemical recurrence was found also for the Cambridge cohort (*P* = 0.022; Fig. 4c).

Performance of the gene score in comparison with clinical markers was evaluated by Cox multivariate analysis in the TCGA-PRAD cohort, where we received robust results and a high statistical power due to the large cohort size. In univariate analysis, both ISUP grade and pathological tumour stage showed prognostic significance together with gene score (HR > 3; *P* < 0.001; Table 2). Moreover, the significance of the gene score was retained in multivariate analysis with a HR of 1.9 (*P* = 0.012; Table 2).

**Table 2:** Cox regression analysis of the TCGA-PRAD cohort (*n* = 491).

	Univariate analysis			Multivariate analysis		
	*P*	HR	95% CI	*P*	HR	95% CI
ISUP	<0.001	4.1	2.3–7.2	0.005	2.4	1.3–4.6
pT	<0.001	3.7	2.1–6.5	0.012	2.2	1.2–4.0
Gene score	<0.001	3.4	2.1–5.5	0.012	1.9	1.2–3.3

Patients were divided into two groups based on a cut-off ISUP ≥ 3 or pT ≥ 3. Gene score was used as a continuous variable. In the multivariate analysis, the same results were obtained for forward and backward selection.

## Discussion

By utilising pimonidazole-stained whole-mount prostatectomy sections, unique hypoxia data of the entire index lesion were available as a reference standard in our study. We provided evidence that the Ragnum-signature reflects hypoxia in this lesion. This evidence is an important, but often lacking, documentation for hypoxia gene signatures, which are commonly based on gene expression responses in cell lines exposed to a selected, reduced oxygen level [6]. We further compared the intra-tumour heterogeneity of the signature with the heterogeneity in pimonidazole-defined hypoxia. Current knowledge of the heterogeneity in different hypoxia measures within prostate tumours is scarce, and our study includes the largest number of patients presented so far. The index lesion may not necessarily represent the most aggressive subpopulation of cancer cells [41]. However, we showed that the hypoxic fraction of this lesion reflects clinical markers of tumour aggressiveness. Moreover, sampling from this lesion by an MRI-directed procedure was shown to be feasible. Our focus on this lesion is therefore of clinical relevance and will facilitate standardisation of the signature as a biomarker.

Pimonidazole-defined hypoxia in a single biopsy from the index lesion showed a weaker association to clinical markers of tumour aggressiveness than when hypoxia in the entire lesion was considered. In accordance with this observation, a large heterogeneity in this hypoxia measure within the lesion was found. The difference in hypoxic fraction between two biopsies from the same lesion was in many cases larger than the separation of patient groups with different clinical markers, and could explain the large number of incorrect classifications of patients in comparison with HF_W-m_. Studies using oxygen electrodes to assess hypoxia as fraction of pO_2_ readings below 5 mmHg have reported an intra-tumour heterogeneity of 39% of the total variability [42]. Although these data were not necessarily obtained only from index lesions, they led to a similar conclusion as ours for another direct hypoxia measure.

The gene score, on the other hand, showed a low intra-tumour heterogeneity, both evaluated by the relative difference between two biopsies in a pair and their correlation. This heterogeneity was also lower than for most of the individual signature genes, in accordance with data for hypoxia gene signatures in other cancer types [43, 44]. In addition, most genes showed a weaker or no correlation with hypoxic fraction in whole-mount sections. These results demonstrate a benefit of using signatures with multiple genes as biomarker. The gene score and hypoxic fraction in whole-mount sections further showed almost equally strong association with tumour aggressiveness. Although these observations, the score classified a considerable number of patients incorrectly according to the pimonidazole-defined measure. Moreover, the incorrect classifications was not due to intra-tumour heterogeneity in the gene score. It is therefore likely that the hypoxia phenotype defined by the score and by pimonidazole differs. In prostate cancer, the pimonidazole-defined hypoxic fraction is mainly determined by the balance between oxygen supply and consumption and thereby physiological parameters like blood perfusion and growth fraction [5]. The gene score, on the other hand, depends on persistent genetic and epigenetic alterations that control transcription, such as mutations, copy number changes and DNA methylations, in addition to an instant, stimulatory effect on gene expression by reduced oxygen concentration. Several genes in our signature are involved in cell cycle and growth control, and a significant, positive correlation between the gene score and expression of the proliferation marker Ki67 has been demonstrated [7]. It is therefore possible that the gene signature captures a phenotype with sustained proliferation under deprived conditions and thus a high tolerance of cancer cells to hypoxia.

The gene score showed prognostic value in three external patient cohorts, validating its association with tumour aggressiveness in our cohort. The external cohorts differed from those used in our previous work to develop the gene signature [7], and included the TCGA-PRAD dataset derived by another gene expression platform than ours. The prognostic value in this cohort was independent of conventional clinical markers, suggesting that the signature may provide information about disease progression that is not covered by current diagnostics. Together, these results strongly support further development of the signature as a biopsy-derived hypoxia biomarker in prostate cancer. In particular, a possible benefit of including samples from other intra-prostatic tumour lesions than the index lesion in calculation of the gene score should be evaluated. Moreover, it would be of interest to explore the added value of combining the signature with medical imaging of hypoxia in a multifactorial treatment decision-support system. Such added value was documented in our recent work on cervical cancer [43]. Our previously developed MR-imaging method visualises hypoxia in prostate tumours as the imbalance between oxygen consumption and supply and provides measures that correlate with pimonidazole-staining in whole-mount sections [5]. The method utilises images acquired during diagnostic examination and can easily be integrated with biopsy sampling from the index lesion. Combining our gene signature with current imaging procedures could thus provide a more complete picture of the hypoxia phenotype and a better informed treatment decision in prostate cancer.

## Supplementary information

Supplementary Methods S1

Supplementary Figures S1-S5

Supplementary Tables S1, S2

Reproducibility checklist

## Data Availability

The gene expression data have been deposited to the Gene Expression Omnibus (GEO) repository (GSE178631).
