# Supplementary material for: A prognostic hypoxia gene signature with low heterogeneity within the dominant tumour lesion in prostate cancer patients
Source: Br J Cancer. 2022 Mar 24;127(2):321–8. doi: 10.1038/s41416-022-01782-x (PMC9296675; doi:10.1038/s41416-022-01782-x)
Supplement: Supplementary file 1 — Supplementary Methods S1 [file 41416_2022_1782_MOESM1_ESM.pdf]

SUPPLEMENTARY METHODS S1

Digital histopathology

Pimonidazole immunostaining was visualized in brown by 3,3-diaminobenzidine (DAB) and counterstained with haematoxylin (blue) for all histological sections. Whole-mount and biopsy sections were imaged at a resolution of 0.46 µm/pixel by a NanoZoomer 2.0-HT and NanoZoomer-XR slide scanner (Hamamatsu, Hamamatsu City, Japan), respectively. In-house made software programs in MatLab were used for image analysis at 3.68 µm/pixel for whole-mount sections and 0.46 µm/pixel for biopsy sections. Colour deconvolution was used to separate out the brown (DAB) colour.

Hypoxic fraction

Hypoxic fraction was quantified by applying two segmentation procedures, as exemplified for a biopsy section in Figure 1. First, background segmentation was performed on the value channel of the hue, saturation, value (HSV) representation of the image, to define the parenchyma tissue to be included in the calculation (Figure 1A-C). Second, segmentation was applied on the brown channel based on the staining intensity defined as hypoxic (Figure 1D-E).

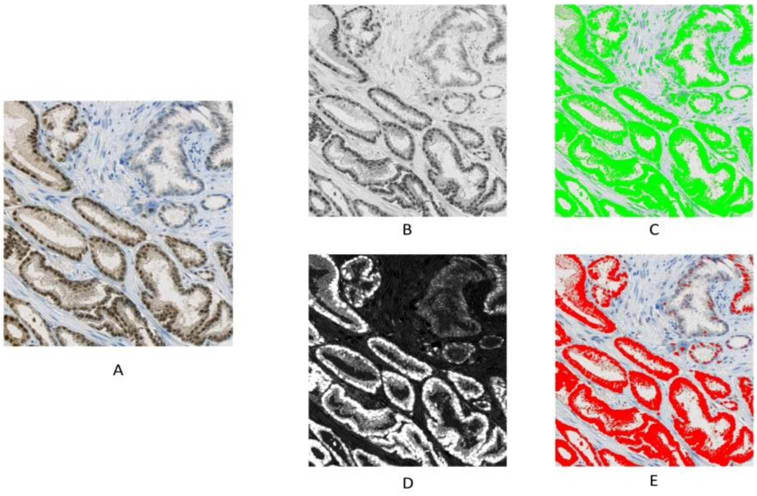

Figure 1: Quantification of hypoxic fraction. (**A**) Pimonidazole-stained tissue section. (**B**) Value channel of the HSV image. (**C**) Tissue included after background segmentation (green), yielding the parenchyma. (**D**) Brown channel image. (**E**) Tissue defined as hypoxic at a segmentation threshold of 0.47 defined in Figure 2C, D (red).

The intensity threshold for segmentation of hypoxic tissue was obtained from optimization against the pathologist's hypoxia score from 100 patients for the whole-mount sections and 38 patients for the biopsy section (Figure 2). This score was generated in previous work from the staining pattern of the tumour parenchyma, as described (1, 2). In short, fractions of nuclear and moderate to strong cytoplasmic immunostaining were determined separately and given a score from 0 to 5 (0: 0%, 1: 1-10%, 2: 11-50%, 3: 51-90%, 4: 91-100%, 5: 100%). The average value of cytoplasmic and nuclear score was used. For whole-mount sections, the optimization led to a segmentation threshold at a the signal intensity of 0.89 (Figure 2A, B). For biopsy sections, the optimal segmentation threshold was at a signal intensity of 0.47 (Figure 2C, D).

The digital hypoxic fraction for whole-mount and biopsy sections, $HF_{W\text{-}m}$ and HF_{Biopsy}, respectively, was calculated as the number of pixels in hypoxic tissue, relative to the number of pixels in the included parenchyma tissue. This measure represents the hypoxic fraction in parenchyma.

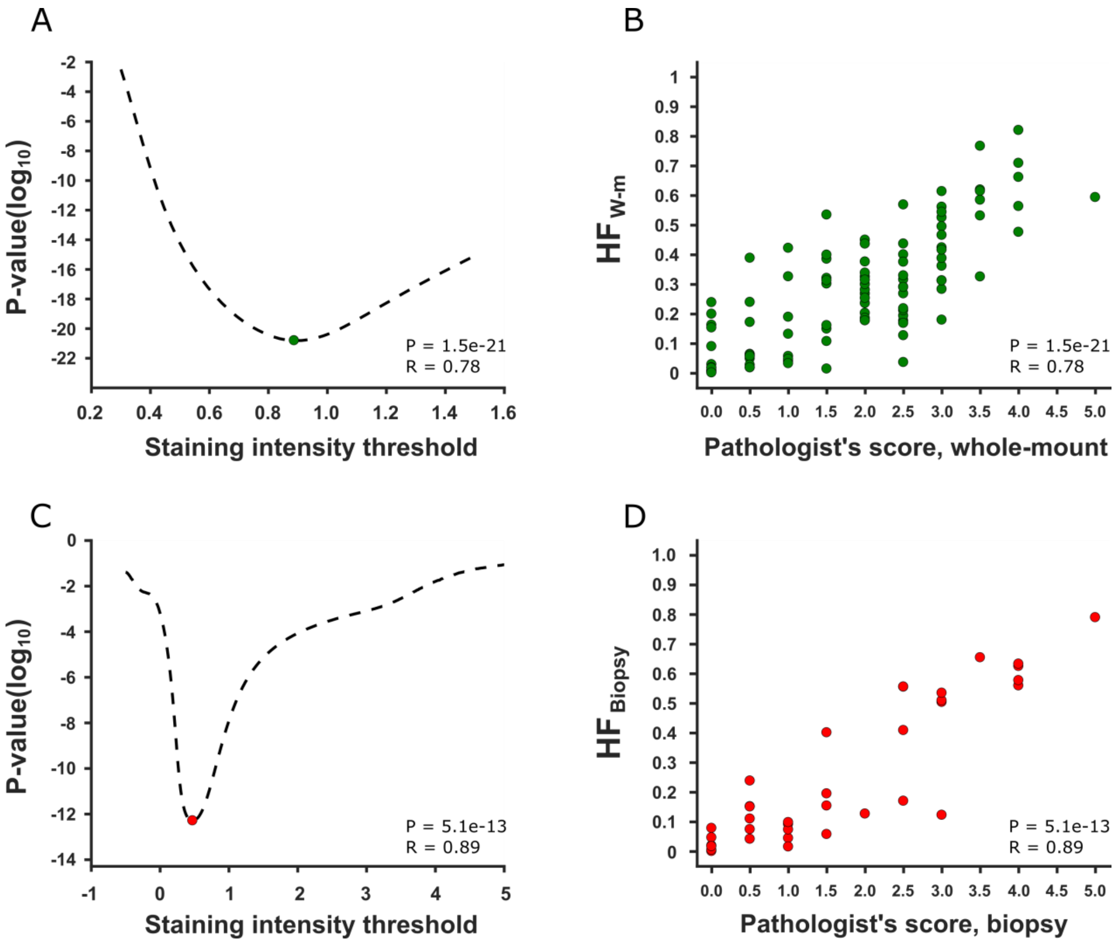

Figure 2: Optimization of segmentation threshold for hypoxia by comparison to the pathologist's score. (**A, C**) P-value from Pearson correlation analysis of the digital hypoxic fraction *versus* pathologist's score at different staining intensity thresholds for segmentation of hypoxic tissue in whole-mount sections (**A**) and biopsy sections (**C**). (**B, D**) Digital hypoxic fraction ($HF_{W\text{-}m}$ and HF_{Biopsy}) *versus* pathologist's score at optimal staining intensity threshold of 0.89 in whole-mount sections (**B**) and 0.47 in biopsy sections (**D**). Pearson correlation coefficient and P-value for the optimal segmentation threshold are indicated.

References

1. Ragnum HB, Vlatkovic L, Lie AK, Axcrona K, Julin CH, Frikstad KM, et al. The tumour hypoxia marker pimonidazole reflects a transcriptional programme associated with aggressive prostate cancer. *Br J Cancer*. 2015;112(2):382-90.

2. Hompland T, Hole KH, Ragnum HB, Aarnes EK, Vlatkovic L, Lie AK, et al. Combined MR imaging of oxygen consumption and supply reveals tumor hypoxia and aggressiveness in prostate cancer patients. *Cancer Research*. 2018;78(16):4774-85.
