# Supplementary material for: A prognostic hypoxia gene signature with low heterogeneity within the dominant tumour lesion in prostate cancer patients
Source: Br J Cancer. 2022 Mar 24;127(2):321–8. doi: 10.1038/s41416-022-01782-x (PMC9296675; doi:10.1038/s41416-022-01782-x)
Supplement: Supplementary file 2 — Supplementary Figures S1-S5 [file 41416_2022_1782_MOESM2_ESM.pdf]

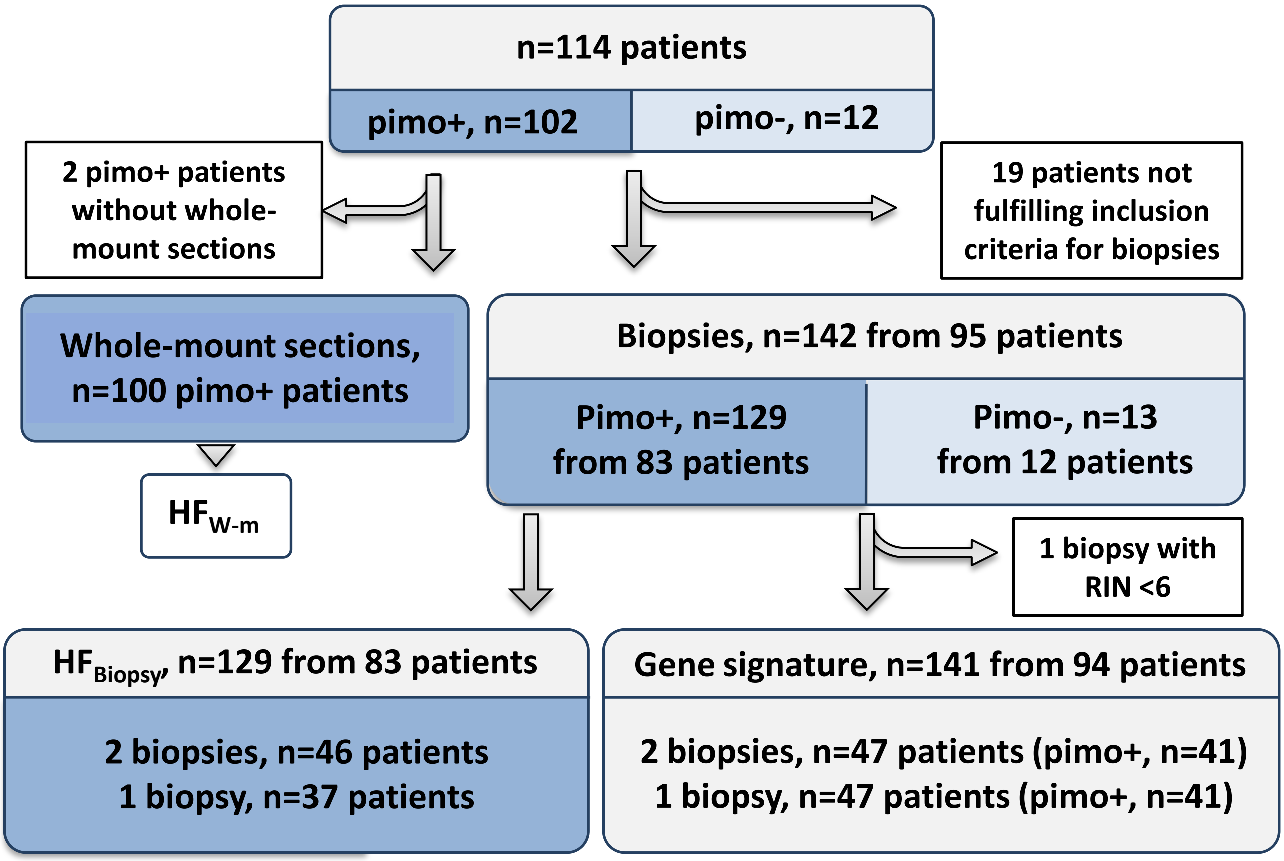

Supplementary Figure S1. Overview of patients and biopsies included in the study. Out of totally 114 patients, 102 patients received pimonidazole (pimo+). Whole-mount sections from 100 of these patients were available and used to assess HF$_{W-m}$. Totally 142 biopsies from 95 out of the 114 patients (83 pimo+, 12 pimo-) were used to assess HF$_{W-m}$ (n=129 biopsies) and gene signature (n=141). Analysis of intratumor heterogeneity was performed in 52 patients with 2 biopsies from the index lesion (HF$_{Biopsy}$, n=46; gene signature, n=47; n=41 patients in common for both assays).

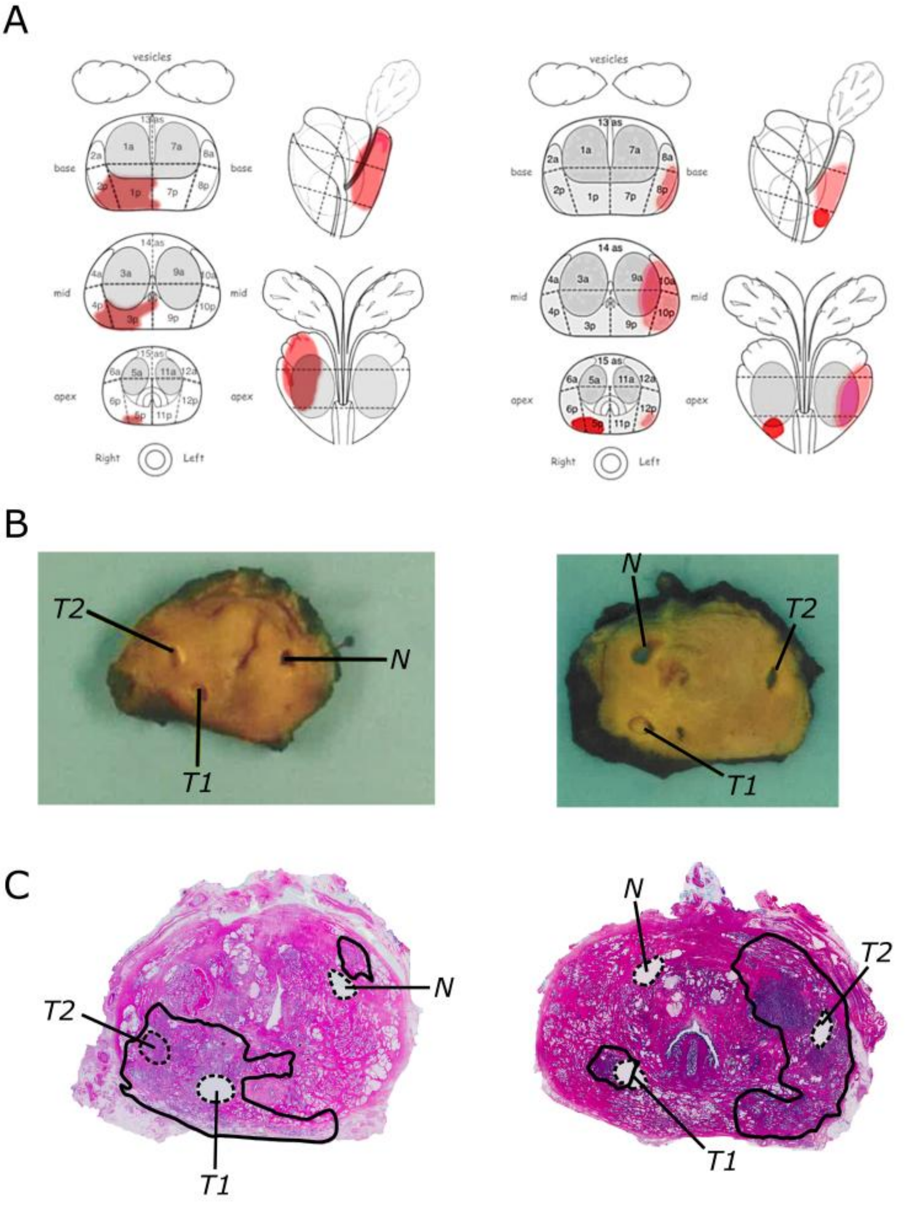

Supplementary Figure S2. Localization of tumour biopsies. The procedure is illustrated for a patient with two biopsies from the index lesion (left) and a patient with one biopsy from the index lesion and one biopsy from another lesion (right). (**A**) MRI reports of the prostate, indicating the site of one (left patient) and two (right patient) intra-prostatic tumour lesions. (**B**) Macro images of the prostatectomy specimens, indicating the site of the punch biopsies. (**C**) HE-stained whole-mount sections of the prostatectomy specimens with the tumour lesions delineated (solid line) and the locations of punch biopsies indicated (dotted line). T1, the biopsy collected first; T2, the second biopsy; N, biopsy collected outside tumour.

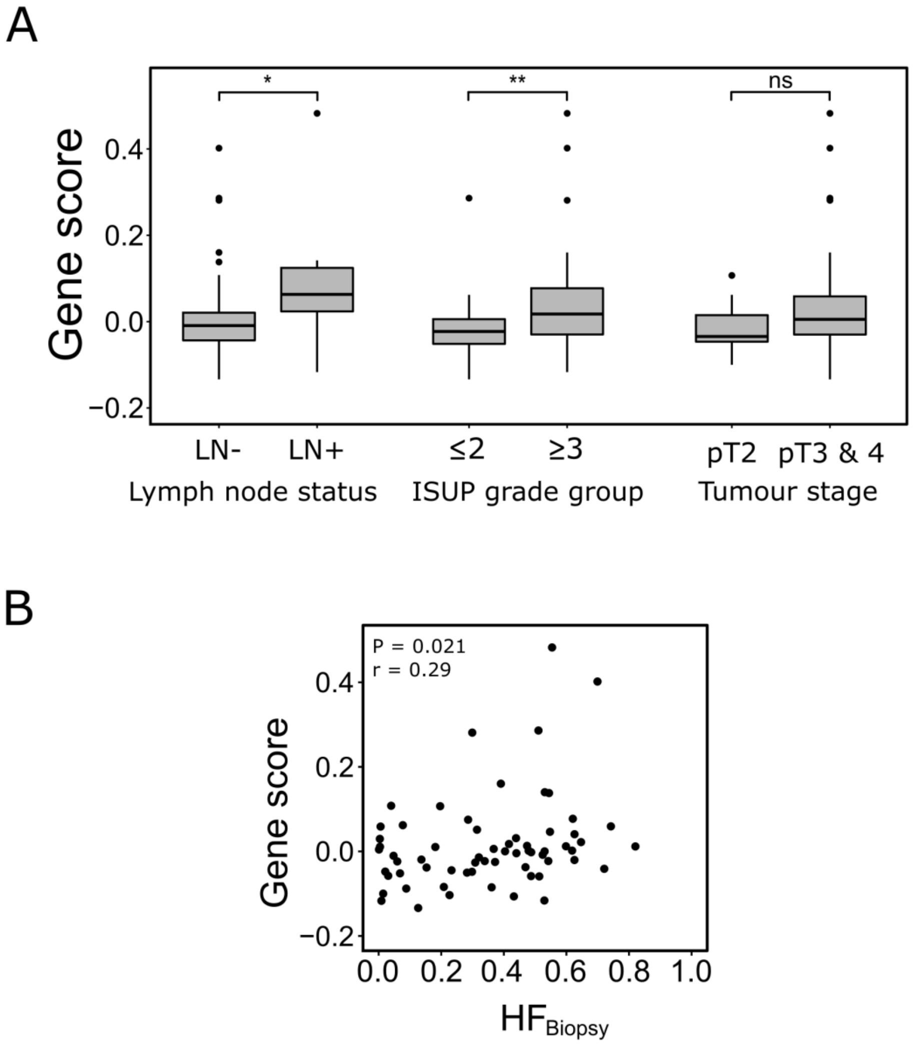

Supplementary Figure S3. Gene score analyses based on biopsies not included in previous work. (**A**) Box plots of gene score for patients with positive and negative lymph node (LN) status (n=74), ISUP grade group of ≤2 and ≥3 (Gleason grade ≤3+4 and ≥4+3) (n=75) and pathological tumour stage T2 and T3 or T4 (n=75). The boxes extend from the first to the third quartile with the median value indicated. Significant difference between groups by Mann-Whitney U-test is indicated; **, P=0.0028, adj P=0.0084; *, P=0.013, adj P=0.0195; ns, not statistically significant P=0.076, adj P=0.076. (**B**) Association between HF_{Biopsy} and gene score for 76 patients with both parameters available. Pearson correlation coefficient (r) and P-value are indicated.

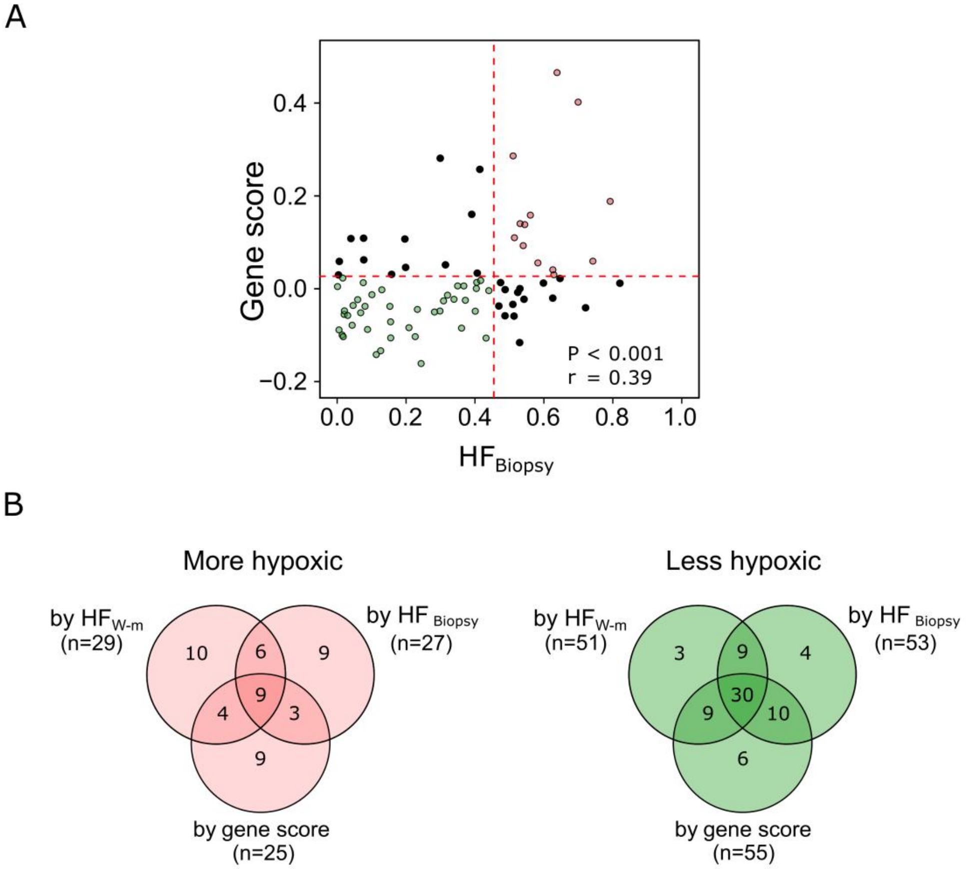

Supplementary Figure S4. Relationship between pimonidazole- and gene-defined hypoxia in biopsies. (**A**) Association between HF_{Biopsy} and gene score for 82 patients with both parameters available. The red dotted lines indicate the separation between more and less hypoxic tumours based on the 67% percentile of the distribution in Figure 2B and D. Pearson correlation coefficient (r) and P-value are indicated. (**B**) Venn-diagram showing the overlap in classification of 80 patients with $HF_{W\text{-}m}$, HF_{Biopsy} and gene score available. The 67% percentiles indicated in (**A**) were used as classification cutoffs for HF_{Biopsy} and gene score, while the 67% percentile of 0.27 was used for $HF_{W\text{-}m}$.

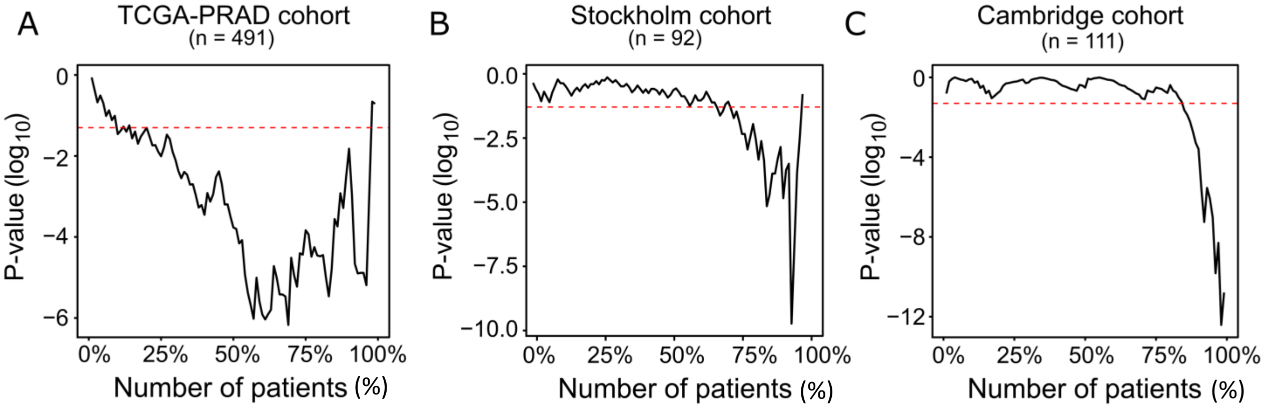

Supplementary Figure S5. Screening of gene score cutoff for patient classification in external data sets. P-value in log rank test *versus* number (%) of patients in the group with a low gene score, based on the TCGA-PRAD cohort (**A**), Stockholm cohort (GSE70769 (**B**), and Cambridge cohort (GSE70768 (**C**). Red line indicates a P-value of 0.05. Number of patients in each cohort is indicated.
