# Supplementary material for: A prognostic hypoxia gene signature with low heterogeneity within the dominant tumour lesion in prostate cancer patients
Source: Br J Cancer. 2022 Mar 24;127(2):321–8. doi: 10.1038/s41416-022-01782-x (PMC9296675; doi:10.1038/s41416-022-01782-x)
Supplement: Supplementary file 3 — Supplementary Tables S1, S2 [file 41416_2022_1782_MOESM3_ESM.pdf]

Supplementary Table S1. Correlation with HF_{W-m} and intratumour heterogeneity for individual genes in the Ragnum-signature.

Gene		Correlation with HF_{W-m}		Intratumour heterogeneity	
Probe ID	SYMBOL	Correlation coefficient (r)	P	Relative difference	Correlation coefficient (r)
ILMN_1708934	ADM	0.16	ns	0.06	0.95
ILMN_1695414	ASF1B	0.18	ns	0.16	0.85
ILMN_1815184	ASPM	0.21	0.063	0.11	0.93
ILMN_2349459	BIRC5	0.28	0.011	0.10	0.90
ILMN_1693145	BUB3	0.16	ns	0.10	0.94
ILMN_1716279	CENPE	0.23	0.042	0.17	0.84
ILMN_1679438	CENPU	0.44	0.000	0.11	0.86
ILMN_1705442	CMTM3	0.15	ns	0.15	0.86
ILMN_1661599	DDIT4	0.28	0.011	0.09	0.92
ILMN_1758629	DONSON	0.18	ns	0.16	0.74
ILMN_1655614	DSP	0.21	0.058	0.12	0.77
ILMN_1779711	DTL	0.15	ns	0.24	0.57
ILMN_2206722	FER1L4	0.29	0.008	0.06	0.94
ILMN_2344971	FOXM1	0.29	0.009	0.13	0.74
ILMN_2347949	G6PD	-0.05	ns	0.14	0.80
ILMN_1659990	HILPDA	0.28	0.012	0.12	0.84
ILMN_1703906	HJURP	0.11	ns	0.08	0.92
ILMN_1681503	MCM2	0.35	0.002	0.10	0.95
ILMN_1659984	MEP1A	0.03	ns	0.17	0.74
ILMN_1659952	MTMR2	0.05	ns	0.13	0.90
ILMN_1693334	P4HA1	0.11	ns	0.10	0.89
ILMN_1706841	PGAM4	0.08	ns	0.12	0.77
ILMN_2366634	PKM	0.15	ns	0.07	0.95
ILMN_1706664	RIMKLA	0.20	0.076	0.12	0.88
ILMN_1776602	RNASE4	-0.10	ns	0.08	0.97
ILMN_1689329	SCD	0.18	ns	0.10	0.85
ILMN_1684620	SPAG4	0.17	ns	0.04	0.99
ILMN_1782331	TDG	0.37	0.001	0.19	0.78
ILMN_1796589	TRIP13	0.25	0.028	0.10	0.91
ILMN_1683120	UNG	0.28	0.013	0.16	0.77
ILMN_2166506	XRCC6	0.14	ns	0.13	0.91
ILMN_2362549	ZWINT	0.09	ns	0.12	0.82

Correlation with HF_{W-m} was assessed by Pearson correlation analysis and based on a single biopy in 80 patients, ns, $P > 0.1$.

Intratumor heterogeneity is based on biopsy pairs in 41 patients. The relative difference was derived as the difference in gene expression level between the two biopsies divided by the overall range (the maximum value minus the minimum value of the 82 biopsies). The correlation was assessed by Pearson correlation analysis of scatter plots where the highest biopsy value in a pair was plotted against the lowest value.

Supplementary Table S2. The Yang-signature in comparison with hypoxic fraction of the index lesion and the Ragnum-signature.

		Hypoxic fraction ($HF_{W\text{-}m}$)		Ragnum signature	
Gene signature	Cancer type	Correlation coefficient (r)	P	Correlation coefficient (r)	P
Yang et al, 2018	Prostate cancer	0.20	0.07	0.04	0.68

A gene score was calculated from the Yang signature as described in Yang et al (2018), using the genes listed below and the gene coefficients listed in Supplementary Table 5 in the Yang-paper.

A gene score was calculated from the Ragnum-signature as described in the present paper, using the genes listed in Supplementary Table S1.

Results from Pearson correlation analysis are listed.

Genes in the Yang-signature

SYMBOL	ENTREZ_GENE_ID
ADAMTS4	9507
ATF3	467
BHLHE40	8553
BTG2	7832
CSRNP1	64651
CYR61	3491
EGR1	1958
EGR2	1959
EGR3	1960
FOSB	2354
FOSL2	2355
GEM	2669
JUNB	3726
KLF10	7071
KLF6	1316
LIF	3976
MCL1	4170
NR4A3	8013
PPP1R15A	23645
RHOB	388
SELE	6401
SIK1	150094
SLC2A14	144195
SLC2A3	6515
SOCS3	9021
THBS1	7057
TIPARP	25976
ZFP36	7538

Reference

Yang, L. *et al.* Development and validation of a 28-gene hypoxia-related prognostic signature for localized prostate cancer. *EBioMedicine* **31**, 182-189 (2018).
